# Evaluation of Antimicrobial Efficacy of various Intracanal Medicaments in Primary Teeth: An *in vivo* Study

**DOI:** 10.5005/jp-journals-10005-1448

**Published:** 2017-02-27

**Authors:** Brahmananda Dutta, Kanika S Dhull, Debasmita Das, PV Samir, Rajnish K Verma, Nipa Singh

**Affiliations:** 1 Professor and Head, Department of Pedodontiocs and Preventive Dentistry, Kalinga Institute of Dental Sciences, KIIT University, Bhubaneswar Odisha, India; 2 Reader, Department of Pedodontiocs and Preventive Dentistry, Kalinga Institute of Dental Sciences, KIIT University, Bhubaneswar Odisha, India; 3 Postgraduate Student, Department of Pedodontiocs and Preventive Dentistry, Kalinga Institute of Dental Sciences, KIIT University, Bhubaneswar Odisha, India; 4 Senior Lecturer, Department of Pedodontiocs and Preventive Dentistry, Kalinga Institute of Dental Sciences, KIIT University, Bhubaneswar Odisha, India; 5 Senior Lecturer, Department of Pedodontiocs and Preventive Dentistry, Kalinga Institute of Dental Sciences, KIIT University, Bhubaneswar Odisha, India; 6 Senior Resident, Department of Microbiology, Kalinga Institute of Medical Sciences, KIIT University, Bhubaneswar Odisha, India

**Keywords:** Calcium hydroxide, Chlorhexidine solution, Triple antibiotic paste.

## Abstract

**Introduction:**

Bacteria and their products play a primary etiological role in the initiation and perpetuation of pulpoperiapical pathosis. Intracanal medication is important for endodontic success as it eliminates microorganisms that persist after chemomechanical preparation.

**Aim:**

To compare antimicrobial efficacy of calcium hydroxide powder, triple antibiotic paste, calcium hydroxide with 2% chlorhexidine solution, and triple antibiotic paste with 2% chlorhexidine solution.

**Materials and methods:**

A total of 48 nonvital primary teeth were included in this study. After access opening first microbiological sample (s1) was collected by using absorbent paper point introducing into canal. Second microbilogical sample (s2) was taken following chemomechanical preparation and the teeth were divided into four groups: Group I: calcium hydroxide (CH) powder with distilled water; group II: CH with 2% chlorhexidine solution; group III: triple antibiotic powder with distilled water; group IV: triple antibiotic paste with 2% chlorhexidine solution. Then the canals were filled with any one group of the medicament and cavity was temporarily sealed with zinc oxide eugenol. After 1 week, a postmedication sample (s3) was collected. Then the canal was filled with Metapex, restored with glass ionomer cement.

**Conclusion:**

From the experiments carried out in this study, with the limitations, an inference can be drawn that a combination of antimicrobial agent used as intracanal medicament is definitely better than single agent like Ca(OH)_2_.

**How to cite this article:**

Dutta B, Dhull KS, Das D, Samir PV, Verma RK, Singh N. Evaluation of Antimicrobial Efficacy of various Intracanal Medicaments in Primary Teeth: An *in vivo* Study. Int J Clin Pediatr Dent 2017;10(3):267-271.

## INTRODUCTION

Dental caries, a progressive disease of the dental hard tissue, if left untreated in its initial stages, may lead to pulpal, periapical, and/or intraradicular infections. There is a similarity in the pathogens found in primary teeth to those in permanent teeth.^1^ Reduction or elimination of the bacterial infection seems to be one of the important factors for the success of endodontic treatment of teeth with necrotic pulp.^2^ Although chemomechani-cal preparation has an important cleaning effect, it does not ensure the complete elimination of microorganisms present in the root canal system of primary teeth owing to their peculiar internal anatomy and the limitations of the instrumentation technique.^3,4^ The remaining viable bacteria left in the root canal may multiply between appointments or following obturation often reaching a pathogenic level and leading to failure.^5^ Hence, a suitable medicament is required to disinfect the root canal system prior to obturation so as to promote healing and prevent recurrence.^6^

Calcium hydroxide (CH) is commonly used in endo-dontics as the intracanal medicament in teeth with necrotic pulp. Its high alkaline pH creates a bacteriostatic environment and the antimicrobial activity is related to its ionic dissociation into calcium and hydroxyl ions and their toxic effects on bacteria, which acts by inhibiting cytoplasmic membrane enzymes with consequent changes in the organic components and in nutrient transport.^7,8^ However, CH is reported to be not much effective against few bacterial strains, especially *Entero-coccus faecalis.^9,10^* Therefore, a combination of agents has been proposed to increase the effectiveness of intracanal medicaments against resistant pathogens. Several other medicaments like chlorhexidine 2% (CHX), either alone or in combination with CH, tripple antibiotic paste (TAP) have been experimented in primary necrotic pulp canals with different degree of success.^11-14^ However, there is a paucity of literature related to efficacy of various combination medication used in primary teeth.

The present study evaluated the antimicrobial efficacy of a new combination medicament (TAP + CH) and compared with other combinations or CH alone against *E. faecalis* in necrotic primary teeth in *in vivo* conditions.

## MATERIALS AND METHODS

Research protocol was approved by the ethical committee of Kalinga Institute of Dental Sciences, KIIT University, Bhubaneswar, Odisha, India, and a written consent was taken prior to the procedure from the parents of children who agreed to participate in the study. Forty-eight children (48 teeth), aged 4 to 6 years, who had open carious lesions in their maxillary or mandibular primary molars with exposure of pulp in clinical diagnosis and radio-graphically revealed interradicular/periapical radiolucen-cies indicating a necrotic pulp and did not have a history of taking any antibiotics for last 3 months, were included in the study. Children were randomly divided into four groups (three positive control and one experimental) depending on the type of intracanal medicament used:

- *Group I:* CH powder with distilled water
- *Group II:* CH powder with 2% CHX gluconate solution
- *Group III:* Triple antibiotic powder with distilled water
- *Group IV:* Triple antibiotic powder with 2% CHX gluconate solution

### Collection of Microbiological Samples

Standard endodontic procedure was performed under rubber dam isolation. Following access cavity preparation, orifices of the larger sized canal (distal in mandibular molars and palatal in maxillary molars) were widened using SX hand files (Dentsply) to fascilate easy entry of paper points deep into the canals. First microbiological samples (S1) were collected by inserting #20 (2%) sterile absorbable paper points, leaving it for about 30 seconds and then transfering into a test tube containing brain heart infusion broth. Second samples (S2) were collected at the end of chemomechanical preparation with SS K-file (Mani) and 2.5% NaOCl and normal saline. Thereafter, one of the above-mentioned medicaments was placed into the root canals using a # 15 K-file as per the allocated group, and the cavity was temporarily sealed with reinforced ZnOE (Kalsinol, DPI, India). Seven days later, canals were reopened, irrigated with normal saline to wash out the medicaments, and third bacteriological samples (S3) were collected. Canals were then obturated with Vitapex and in subsequent appointments restored with glass ionomer cement (GC Fuji IX, Japan) followed by cementation of SS crown (Kids crown, India).

### Microbiological Procedures

Test tubes containing microbiological samples were pre-incubated for 30 minutes at 37°C and shaken vigorously in a vertex mixer for 60 seconds. Serial 10-fold dilutions were made up to 1:10^6^ in 1% sterile saline. From the serial dilutions, 0.1 mL was transferred on to blood agar and MacConkey agar plates. The plates were incubated for 24 hours. All plates were counted for colony-forming units (CFU)/mL after 24 hours. The organisms *(E. faecalis)* were identified using Gram staining, catalase production, colony morphology on blood agar by using bile esculin agar.

Statistical analysis was carried out using Statistical Package for the Social Sciences version 20 software program (SPSS Inc, Chicago, IL, USA) and using one-way analysis of variance and Turkey’s honest significant difference and t-tests.

## RESULTS

Out of 48 necrotic primary teeth studied, *E. faecalis* was isolated from 37 teeth (75%) and results were evaluated only for those 37 teeth.

**Fig. 1:**
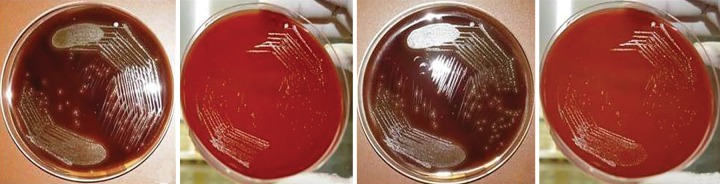
S1 samples (CFU/mL) of different groups

**Fig. 2:**
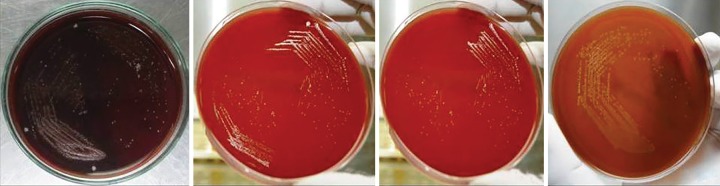
S2 samples (CFU/mL) of different groups

**Fig. 3:**
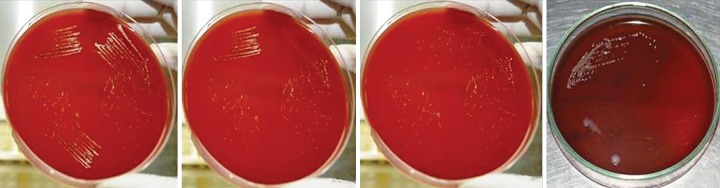
S3 samples (CFU/mL) of different groups

*Enterococcus faecalis* counts obtained in the necrosed root canals prior to instrumentation (S1) between the groups were comparable and nonsignificant (p > 0.05) (Table 1 and Fig. 1). In the second microbiological samples (S2), which was taken immediately after chemomechani-cal preparation, a statistically significant reduction in *E. faecalis* count (p < 0.001) was observed in all groups (Fig. 2). However, while those values were compared between the groups, these differences were not significant. All the four groups exhibited a significant reduction in *E. faecalis* count (p < 0.001) in third samples (S3) collected 7 days following intracanal medication, compared with their respective values in S2 (Table 2 and Fig. 3). Maximum bacterial inhibition was observed in experimental group (group IV) and minimum in CH group (group I). Intra-group comparison at S3 level (Table 2) revealed a highly significant difference in reduction of *E. faecalis* count between groups I and II, groups I and III, and groups I and IV (p < 0.001). However, the effect of intracanal medicament in reducing *E. faecalis* count was not significant between groups II and III and groups II and IV.

## DISCUSSION

It should be perceived that majority of teeth indicated for pulp therapy are irreversibly inflammed and only a small percentage of cases exhibit infected or necrotic pulp. The rationale of using intracanal medicament in primary teeth with infected canals is based on the concept that ribbon-shaped pulp spaces cannot be completely disinfected following chemomechanical preparation. With the use of intracanal medicaments prior to obturation of root canals, the predictability and prognosis of endodontic success could be enhanced by many fold. *Enterococcus faecalis* is chosen as test species because of its isolation in therapy-resistant apical infection. Besides, *in vitro* studies have shown its penetration into deeper dentinal tubules^4,15^ which can be implicated for recurrence of infection.

**Table 1:** **Table 1:** Sample distribution and mean standard deviation of *E. faecalis* count at different levels

*Serial no*		*The mean (SD) of E. faecalis count (CFU/mL) before instrumentation (S1)*		*The mean (SD) of E. faecalis count (CFU/mL) after instrumentation (S2)*		*The mean (SD) of E. faecalis count (CFU/mL) after 7 days intracanal medication (S3)*	
Group I n = 9		1.85 (± 0.59)×10^5^		7.73 (± 2.67)×10^3^		5.03 (± 1.30)×10^1^	
Group II n = 10		1.84 (± 0.63)×10^5^		6.59 (± 1.71)×10^3^		3.15 (± 0.59)×10^1^	
Group III n = 9		2.03 (± 0.48)×10^5^		8.78 (± 1.10)×10^3^		3.52 (± 0.54)×10^1^	
Group IV n = 9		2.00 (± 0.58)×10^5^		7.89 (± 1.91)×10^3^		2.65 (± 0.73)×10^1^	

SD: Standard deviation

**Table 2:** **Table 2:** Intragroup comparison of *E. faecalis* count at S3 level

*Dependent variable*		*Groups*		*Subgroups*		*Mean difference*		*Std. error*		*Significance*	
		I		II		19.21		3.77		0.00***	
				III		15.40		3.77		0.00***	
				IV		24.77		3.77		0.00***	
		II		I		–19.21		3.77		0.00***	
				III		–3.81		3.77		0.74	
S3				IV		5.56		3.77		0.46	
		III		I		–15.40		3.77		0.00***	
				II		3.81		3.77		0.74	
				IV		9.37		3.77		0.08*	
		IV		I		–24.77		3.77		0.00***	
				II		–5.56		3.76		0.46	
				III		–9.37		3.77		0.08*	

*Significant; ***Highly significant

The first reported use of antibiotics as an intracanal medicament could be referred to 1951, when Grossman introduced a polyantibiotic paste known as PBSC.^15,16^ Thereafter, medicaments like Ca(OH)_2_, CHX (2%), triple antibiotic paste, and propolis have been experimented with varied degree of success.^14,16-18^ However, with the change in the ecosystem of infected root canals, and probable mutation of the existing bacterial gene, it is unlikely that a single antimicrobial agent could result in effective sterilization of the canals. Therefore, a combination of these agents has been experimented, which will have an additive/synergistic effect to address these diverse microflora.

In the present study, a new combination (TAP + CHX) was used to assess its efficacy on *E. faecalis* count inhibition and was compared with CH alone or combination of agents, which have already been experimented. As anticipated, all the four groups displayed statistically significant reduction in bacterial count between S1 and S2 and S2 and S3, confirming the effectiveness of chemome-chanical preparation as well as intracanal medicaments in reducing *E. faecalis* counts. Following intracanal medicament at S3 level , combination of agents (groups II and III) exhibited better effectiveness in reducing *E. faecalis* count than CH alone (group I) (p < 0.001). The rationale of using combination of antimicrobial agents to achieve synerg-estic effect has also been corroborated by the results of other studies.^20-23^ Contrary to this, while comparison was made between groups II, III, and IV, group IV displayed maximum inhibition against *E. faecalis* count than groups III and 4. However, none of the group was significantly better efficacious than the other groups (p > 0.05). Nevertheless, some *in vitro* studies demonstrated that CHX gel alone has a greater antimicrobial effect than the combination.^17,19,24-27^

For any intracanal medicament, to exhibit its biological effect either alone or in combination, several factors like drug concentration, drug interaction, better compaction into the canals, and of course the virulence of the intracanal microorganisms should be relied on. Besides, a difference in individual technique should not also be ignored. Although our study did not reveal that combination of triple antibiotic powder with 2% CHX is not better than other combinations, further studies with similar type of experiment are required in future to support or oppose our findings.

## CONCLUSION

From the experiment carried out in this study, with the limitations, an inference can be drawn that a combination of antimicrobial agents used as intracanal medicament is definitely better than a single agent like Ca(OH)_2_. Any combinations commonly used is equally effective against *E. faecalis,* a resistant intracanal bacteria commonly seen in an infected or necrotic primary root canals.
